# Dimethyl 2,2-bis­(2-cyano­ethyl)malonate

**DOI:** 10.1107/S1600536808005850

**Published:** 2008-04-16

**Authors:** Guo-Wei Wang, Ling-Hua Zhuang, Wen-Yuan Wu, Jin-Tang Wang

**Affiliations:** a Department of Applied Chemistry, College of Science, Nanjing University of Technology, Nanjing 210009, People’s Republic of China

## Abstract

The asymmetric unit of the title compound, C_11_H_14_N_2_O_4_, contains one half-mol­ecule; a twofold rotation axis passes through the central C atom. Inter­molecular C—H⋯N hydrogen bonds link the mol­ecules into a one-dimensional supra­molecular structure.

## Related literature

For general background, see: Kim *et al.* (2001 ▶); Chetia *et al.* (2004 ▶); Zhang *et al.* (2004 ▶); Ranu & Banerjee (2005 ▶). For bond–length data, see: Allen *et al.* (1987 ▶).
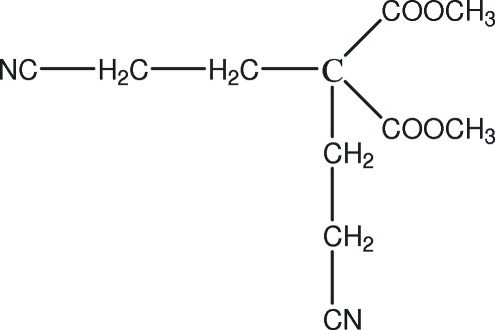


## Experimental

### 

#### Crystal data

C_11_H_14_N_2_O_4_
*M* *_r_* = 238.24
Monoclinic, 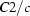

*a* = 13.071 (3) Å
*b* = 8.5060 (17) Å
*c* = 10.914 (2) Å
β = 90.55 (3)°
*V* = 1213.4 (4) Å^3^
*Z* = 4
Mo *K*α radiation
μ = 0.10 mm^−1^
*T* = 293 (2) K
0.40 × 0.30 × 0.20 mm

#### Data collection

Enraf–Nonius CAD-4 diffractometer
Absorption correction: ψ scan (North *et al.*, 1968 ▶) *T*_min_ = 0.961, *T*_max_ = 0.975
1140 measured reflections
1091 independent reflections
860 reflections with *I* > 2σ(*I*)
*R*_int_ = 0.048
3 standard reflections every 200 reflections intensity decay: none

#### Refinement

*R*[*F* ^2^ > 2σ(*F* ^2^)] = 0.065
*wR*(*F* ^2^) = 0.155
*S* = 0.99
1091 reflections
78 parameters
H-atom parameters constrained
Δρ_max_ = 0.21 e Å^−3^
Δρ_min_ = −0.24 e Å^−3^

### 

Data collection: *CAD-4 Software* (Enraf–Nonius, 1989 ▶); cell refinement: *CAD-4 Software*; data reduction: *XCAD4* (Harms & Wocadlo, 1995 ▶); program(s) used to solve structure: *SHELXS97* (Sheldrick, 2008 ▶); program(s) used to refine structure: *SHELXL97* (Sheldrick, 2008 ▶); molecular graphics: *SHELXTL* (Sheldrick, 2008 ▶); software used to prepare material for publication: *SHELXTL*.

## Supplementary Material

Crystal structure: contains datablocks global, I. DOI: 10.1107/S1600536808005850/rk2080sup1.cif

Structure factors: contains datablocks I. DOI: 10.1107/S1600536808005850/rk2080Isup2.hkl

Additional supplementary materials:  crystallographic information; 3D view; checkCIF report

## Figures and Tables

**Table 1:** Hydrogen-bond geometry (Å, °)

*D*—H⋯*A*	*D*—H	H⋯*A*	*D*⋯*A*	*D*—H⋯*A*
C6—H6*B*⋯N1^i^	0.96	2.57	3.494 (5)	161

Symmetry code: (i) 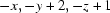.

**Fig. 1.**
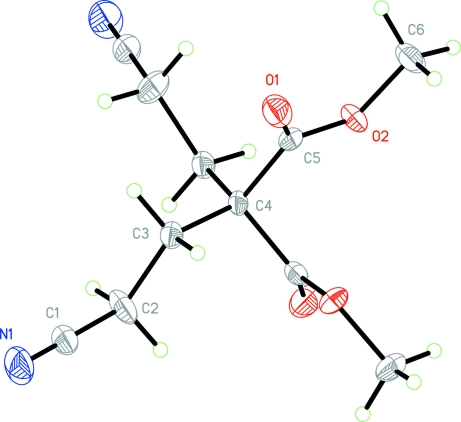
A view of the molecular structure of I showing the atom–numbering scheme. Displacement ellipsoids are drawn at 30% probability level. H atoms are presented as a spheres of arbitrary radius.

**Fig. 2.**
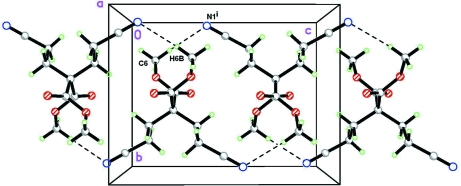
The 1–D supramolecular structure developed by C—H···N hydrogen bonds (dashed lines) [Symmetry codes: (i) -x, 2 - y, 1 - z].

## supplementary crystallographic information

### Comment

Dicarbonyl compounds represent an important class of starting materials to increase the carbon number of organic compounds (Kim *et al.*, 2001). Some dicarbonyl compounds are useful for the synthesis of enantiomerically pure alcohols (Chetia *et al.*, 2004).

Many dicarbonyl compounds have been synthesized with "Michael Addition" method using diethy malonate as starting compound, but only a few "Michael Addition" diadducts were synthesized under normal condition (Zhang *et al.*, 2004; Ranu & Banerjee, 2005). We are focusing our synthetic and structure studies on new products of "Michael Addition" diadducts from dicarbonyl compounds. We here report the crystal structure of the title compound (I).

The atom–numbering scheme of I is shown in Fig. 1, and all bond lengths and angles are within normal ranges (Allen *et al.*, 1987). The asymmetric unit contains one half–molecule, and C4 lies on the twofold rotation axis vertical to *ac* plane, which generates the other half–molecule. An intermolecular C—H···N hydrogen bond (table and Fig. 2) helps to establish the 1–*D* supramolecular structure.

### Experimental

Dimethyl malonate (50 mmol) was dissolves in *n*–hexane (20 ml), then anhydrous potassium carbonate (100 mmol) and tetrabutylammonium bromide (1 g) was added. Finally acrylonitrile (100 mmol) was slowly dropped to the solution above. The resulting mixture was refluxed for 12 h, and 100 ml water was added to the mixture and the organic layer was dried with magnesium sulfate and vacuumed to removed the solvent. Then the crude compound I was obtained. It was crystallized from ethyl acetate (15 ml). Crystals of I suitable for *X*–ray diffraction were obtained by slow evaporation of an alcohol solution. ^1^H NMR (CDCl_3_, δ, p.p.m.) 3.83 (s, 6H), 2.47 (t, 4H), 2.26 (t, 4H).

### Refinement

All H atoms were positioned geometrically, with C—H = 0.96 and 0.97Å for methyl and methylene H atoms, and constrained to ride on their parent atoms, with *U*_iso_(H) = *xU*_eq_(C), where *x* = 1.5 for methyl H and *x* = 1.2 for methylene H atoms.

### Crystal data

**Table:**

C_11_H_14_N_2_O_4_	*F*_000_ = 504
*M**_r_* = 238.24	*D*_x_ = 1.304 Mg m^−^^3^
Monoclinic, *C*2/*c*	Mo *K*α radiation λ = 0.71073 Å
Hall symbol: -C 2yc	Cell parameters from 25 reflections
*a* = 13.071 (3) Å	θ = 10–14º
*b* = 8.5060 (17) Å	µ = 0.10 mm^−^^1^
*c* = 10.914 (2) Å	*T* = 293 (2) K
β = 90.55 (3)º	Block, colourless
*V* = 1213.4 (4) Å^3^	0.40 × 0.30 × 0.20 mm
*Z* = 4	

### Data collection

**Table:**

Enraf–Nonius CAD-4 diffractometer	*R*_int_ = 0.048
Radiation source: Fine–focus sealed tube	θ_max_ = 25.2º
Monochromator: Graphite	θ_min_ = 2.9º
*T* = 293(2) K	*h* = −15→15
ω/2θ scans	*k* = 0→10
Absorption correction: ψ scan(North *et al.*, 1968)	*l* = 0→12
*T*_min_ = 0.961, *T*_max_ = 0.975	3 standard reflections
1140 measured reflections	every 200 reflections
1091 independent reflections	intensity decay: none
860 reflections with *I* > 2σ(*I*)	

### Refinement

**Table:**

Refinement on *F*^2^	Secondary atom site location: Difmap
Least-squares matrix: Full	Hydrogen site location: Geom
*R*[*F*^2^ > 2σ(*F*^2^)] = 0.065	H-atom parameters constrained
*wR*(*F*^2^) = 0.155	*w* = 1/[σ^2^(*F*_o_^2^) + (0.0591*P*)^2^ + 3.2284*P*] where *P* = (*F*_o_^2^ + 2*F*_c_^2^)/3
*S* = 0.99	(Δ/σ)_max_ < 0.001
1091 reflections	Δρ_max_ = 0.21 e Å^−^^3^
78 parameters	Δρ_min_ = −0.24 e Å^−^^3^
Primary atom site location: Direct	Extinction correction: None

### Special details

**Table:**

Geometry. All s.u.'s (except the s.u. in the dihedral angle between two l.s. planes) are estimated using the full covariance matrix. The cell s.u.'s are taken into account individually in the estimation of s.u.'s in distances, angles and torsion angles; correlations between s.u.'s in cell parameters are only used when they are defined by crystal symmetry. An approximate (isotropic) treatment of cell s.u.'s is used for estimating s.u.'s involving l.s. planes.
Refinement. Refinement of *F*^2^ against ALL reflections. The weighted *R*–factor w*R* and goodness of fit *S* are based on *F*^2^, conventional *R*–factors *R* are based on *F*, with *F* set to zero for negative *F*^2^. The threshold expression of *F*^2^ > 2σ(*F*^2^) is used only for calculating *R*–factors(gt) *etc*. and is not relevant to the choice of reflections for refinement. *R*–factors based on *F*^2^ are statistically about twice as large as those based on *F*, and *RR*–factors based on ALL data will be even larger.

### Fractional atomic coordinates and isotropic or equivalent isotropic displacement parameters (Å^2^)

**Table:**

	*x*	*y*	*z*	*U*_iso_*/*U*_eq_	
N1	−0.1143 (3)	0.5595 (3)	0.4119 (3)	0.0730 (10)	
C1	−0.1098 (2)	0.6248 (3)	0.5039 (3)	0.0471 (7)	
O1	0.15852 (15)	1.0070 (2)	0.6705 (2)	0.0516 (6)	
O2	0.09191 (13)	1.1101 (2)	0.84034 (16)	0.0402 (5)	
C2	−0.1041 (3)	0.7072 (4)	0.6228 (3)	0.0589 (9)	
H2A	−0.1056	0.6311	0.6890	0.071*	
H2B	−0.1628	0.7760	0.6312	0.071*	
C3	−0.00519 (19)	0.8043 (3)	0.6315 (2)	0.0333 (6)	
H3A	−0.0013	0.8737	0.5612	0.040*	
H3B	0.0532	0.7340	0.6293	0.040*	
C4	0.0000	0.9032 (4)	0.7500	0.0301 (8)	
C5	0.09365 (19)	1.0115 (3)	0.7444 (2)	0.0309 (6)	
C6	0.1753 (2)	1.2212 (4)	0.8494 (3)	0.0481 (8)	
H6A	0.1667	1.2853	0.9209	0.072*	
H6B	0.1756	1.2867	0.7778	0.072*	
H6C	0.2390	1.1653	0.8555	0.072*	

### Atomic displacement parameters (Å^2^)

**Table:**

	*U*^11^	*U*^22^	*U*^33^	*U*^12^	*U*^13^	*U*^23^
N1	0.0893 (16)	0.0651 (16)	0.0628 (19)	0.0048 (16)	−0.0436 (17)	−0.0172 (15)
C1	0.0547 (18)	0.0469 (14)	0.0532 (17)	−0.0015 (14)	−0.0192 (13)	−0.0050 (14)
O1	0.0430 (12)	0.0432 (12)	0.0585 (14)	−0.0076 (9)	0.0028 (10)	−0.0102 (10)
O2	0.0498 (10)	0.0442 (10)	0.0465 (11)	−0.0111 (8)	−0.0098 (8)	−0.0088 (8)
C2	0.0571 (18)	0.0484 (17)	0.0582 (13)	−0.0162 (16)	−0.0205 (16)	−0.0162 (15)
C3	0.0404 (14)	0.0479 (12)	0.0355 (13)	0.0018 (11)	−0.0067 (10)	−0.0007 (10)
C4	0.0476 (19)	0.0472 (16)	0.0355 (18)	−0.0017 (10)	−0.0025 (14)	0.0006 (10)
C5	0.0421 (13)	0.0469 (13)	0.0344 (13)	0.0058 (10)	−0.0093 (10)	0.0032 (10)
C6	0.0477 (17)	0.0452 (16)	0.0549 (18)	−0.0162 (14)	−0.0138 (13)	−0.0046 (13)

### Geometric parameters (Å, °)

**Table:**

N1—C1	1.149 (4)	C3—H3A	0.9700
C1—C2	1.476 (4)	C3—H3B	0.9700
O1—C5	1.177 (3)	C4—C5	1.534 (3)
O2—C5	1.341 (3)	C4—C5^i^	1.534 (3)
O2—C6	1.445 (3)	C4—C3^i^	1.544 (3)
C2—C3	1.537 (4)	C6—H6A	0.9600
C2—H2A	0.9700	C6—H6B	0.9600
C2—H2B	0.9700	C6—H6C	0.9600
C3—C4	1.544 (3)		
			
N1—C1—C2	179.4 (4)	C5—C4—C3	108.85 (13)
C5—O2—C6	116.3 (2)	C5^i^—C4—C3	109.39 (13)
C1—C2—C3	110.1 (3)	C5—C4—C3^i^	109.39 (13)
C1—C2—H2A	109.6	C5^i^—C4—C3^i^	108.85 (13)
C3—C2—H2A	109.6	C3—C4—C3^i^	113.9 (3)
C1—C2—H2B	109.6	O1—C5—O2	125.0 (2)
C3—C2—H2B	109.6	O1—C5—C4	126.0 (2)
H2A—C2—H2B	108.1	O2—C5—C4	108.96 (19)
C2—C3—C4	112.0 (2)	O2—C6—H6A	109.5
C2—C3—H3A	109.2	O2—C6—H6B	109.5
C4—C3—H3A	109.2	H6A—C6—H6B	109.5
C2—C3—H3B	109.2	O2—C6—H6C	109.5
C4—C3—H3B	109.2	H6A—C6—H6C	109.5
H3A—C3—H3B	107.9	H6B—C6—H6C	109.5
C5—C4—C5^i^	106.2 (3)		
			
C1—C2—C3—C4	175.4 (2)	C5^i^—C4—C5—O1	−126.7 (3)
C2—C3—C4—C5	−173.0 (2)	C3—C4—C5—O1	−9.0 (3)
C2—C3—C4—C5^i^	−57.4 (3)	C3^i^—C4—C5—O1	116.0 (3)
C2—C3—C4—C3^i^	64.63 (19)	C5^i^—C4—C5—O2	55.37 (14)
C6—O2—C5—O1	2.0 (4)	C3—C4—C5—O2	173.02 (18)
C6—O2—C5—C4	180.0 (2)	C3^i^—C4—C5—O2	−61.9 (2)

Symmetry codes: (i) −*x*, *y*, −*z*+3/2.

### Hydrogen-bond geometry (Å, °)

**Table:**

*D*—H···*A*	*D*—H	H···*A*	*D*···*A*	*D*—H···*A*
C6—H6B···N1^ii^	0.96	2.57	3.494 (5)	161

Symmetry codes: (ii) −*x*, −*y*+2, −*z*+1.
